# Identification of hub genes with diagnostic values in pancreatic cancer by bioinformatics analyses and supervised learning methods

**DOI:** 10.1186/s12957-018-1519-y

**Published:** 2018-11-14

**Authors:** Chunyang Li, Xiaoxi Zeng, Haopeng Yu, Yonghong Gu, Wei Zhang

**Affiliations:** 1 West China Biomedical Big Data Center, West China Hospital, Sichuan University, Chengdu, China; 2 Medical Big Data Center, Sichuan University, Chengdu, China

**Keywords:** Pancreatic cancer, Bioinformatics analysis, Differentially expressed genes, Hub genes, Diagnosis

## Abstract

**Background:**

Pancreatic cancer is one of the most lethal tumors with poor prognosis, and lacks of effective biomarkers in diagnosis and treatment. The aim of this investigation was to identify hub genes in pancreatic cancer, which would serve as potential biomarkers for cancer diagnosis and therapy in the future.

**Methods:**

Combination of two expression profiles of GSE16515 and GSE22780 from Gene Expression Omnibus (GEO) database was served as training set. Differentially expressed genes (DEGs) with top 25% variance followed by protein-protein interaction (PPI) network were performed to find candidate genes. Then, hub genes were further screened by survival and cox analyses in The Cancer Genome Atlas (TCGA) database. Finally, hub genes were validated in GSE15471 dataset from GEO by supervised learning methods *k*-nearest neighbor (*k*NN) and random forest algorithms.

**Results:**

After quality control and batch effect elimination of training set, 181 DEGs bearing top 25% variance were identified as candidate genes. Then, two hub genes, *MMP7* and *ITGA2*, correlating with diagnosis and prognosis of pancreatic cancer were screened as hub genes according to above-mentioned bioinformatics methods. Finally, hub genes were demonstrated to successfully differ tumor samples from normal tissues with predictive accuracies reached to 93.59 and 81.31% by using *k*NN and random forest algorithms, respectively.

**Conclusions:**

All the hub genes were associated with the regulation of tumor microenvironment, which implicated in tumor proliferation, progression, migration, and metastasis. Our results provide a novel prospect for diagnosis and treatment of pancreatic cancer, which may have a further application in clinical.

**Electronic supplementary material:**

The online version of this article (10.1186/s12957-018-1519-y) contains supplementary material, which is available to authorized users.

## Background

Pancreatic cancer is one of the most lethal tumors due to the poor prognosis, and now it is the fourth or fifth most common causes of cancer mortality in developed countries [1]. And it is estimated that by the year 2020, pancreatic cancer would move to the second leading cause of death [2]. Although some advances in understanding the molecular mechanisms of pancreatic cancer have been achieved, there still exist difficulties in early diagnosis due to non-specific symptoms and lacking effective testing identification, making it usually found in its late stage [3]. Until now, 1-year survival in pancreatic cancer patients is still not significantly improved [4], and the 5-year survival is less than 10% [5].

Numerous studies have focused on the investigation of biomarkers and molecular mechanisms of pancreatic cancers, and it is demonstrated that accumulated mutations in genes like oncogene *Kras*, and tumor-suppressor genes including *P16* as well as *TP53* resulted in the occurrence of pancreatic cancer [4]. One study performed the whole-genome sequencing and copy number variation (CNV) analyses showed that several genes including *TP53*, *SMAD4*, *CDKN2A*, *ARID1A*, *ROBO2*, *PREX2*, and *KDM6A* were disrupt resulting from chromosomal rearrangements in pancreatic ductal adenocarcinomas patients [6]. Molecular mechanisms researches demonstrated that overexpression of protein-coupled receptor GPR87 enhanced pancreatic cancer aggressiveness by activating NF-κB signaling pathway [7]. Moreover, Zhong and colleges have found that functional P38 MAPK activity contributed to overall survival through suppressing JNK signaling in pancreatic cancer [8]. In addition, aberrant expressions of some microRNAs have emerged as an important hallmark of cancer recently [9]. It was reported that microRNA-21 was overexpressed in pancreatic cancer, and could serve as a potential predictor of survival [10]. One study has found that miR-506 facilitated pancreatic cancer progression and chemoresistance via SPHK1/Akt/NF-κB signaling pathway [11]. Another study demonstrated that suppressing microRNA-34 expression downregulated Bcl-2 and Notch1/2 in pancreatic cancer cells, as well as significantly inhibited cell growth and invasion, induced apoptosis and G1 and G2/M arrest in cell cycle, and sensitized the cells to chemotherapy and radiation [12].

However, traditional experimental methods as mentioned above could only identify single gene or a few genes at once, which limits large-scale investigation of hub genes and pathways in the systematic biology level. Development of microarray and sequencing technologies provides better methods for biomarker screening and molecular mechanism discovery in cancer research. Recent years with the accessibility of multi-omics database like Gene Expression Omnibus (GEO) [13] as well as The Cancer Genome Atlas (TCGA) [14] and so on, it is now possible to acquire multi-sample data and compare cancer profiles with normal profiles in multiple omics dimensions. On one hand, omics data in multiple dimensions leading to the system biology- and/or network-based approach, which could better understand the dysregulated molecular mechanisms in cancer development and progression [15]. On the other hand, biology- and/or network-based method can not only identify critical genes but also can detect corresponding pathways and/or interactive network, which may provide better insights into molecular mechanisms investigation than dysregulated gene analysis individually [16]. For example, *Kras* was proved to be the most frequently mutated gene in pancreatic ductal adenocarcinoma [17], and the mutation of *Kras* was a hallmark of pancreatic cancer [18]. However, inhibitors targeting *Kras* gene were largely unsuccessful, while some omics-based strategies targeting *Kras* correlated pathways and interactive genes were proved to bear better therapeutic effects than targeting *Kras* individually [19].

To date, diagnosis of pancreatic cancer is mainly based on clinical signs and pathology confirmation. However, the specific symptoms and pathological imagines may only be detected unambiguously at the late stage of pancreatic cancer, which may lead to a limited therapies and poor prognosis. This raises an urgent need for the development of reliable biomarkers which can effectively differ tumor from normal tissues based on analyses of gene expression profiles. Herein, in order to identify novel diagnostic predictors and molecular markers, we integrated two microarray datasets from GEO database, and 11 candidate genes significantly differentially expressed between tumor and normal samples were screened by bioinformatics analyses. Then two hub genes, matrix metallopeptidase 7 (*MMP7*) and integrin, alpha 2 (*ITGA2*), were further identified by survival and cox analyses in TCGA database. These two hub genes were validated in another expression profile from GEO database, demonstrating that these hub genes can successfully differ normal tissues from tumor samples. The predictive accuracies of *k*-nearest neighbor (*k*NN) and random forest algorithms were almost 94% and almost 82%, respectively. Results in our study may provide an auxiliary evidence of pancreatic cancer diagnosis and therapy in the future.

## Methods

### Data collection and preprocessing

**Fig. 1:**
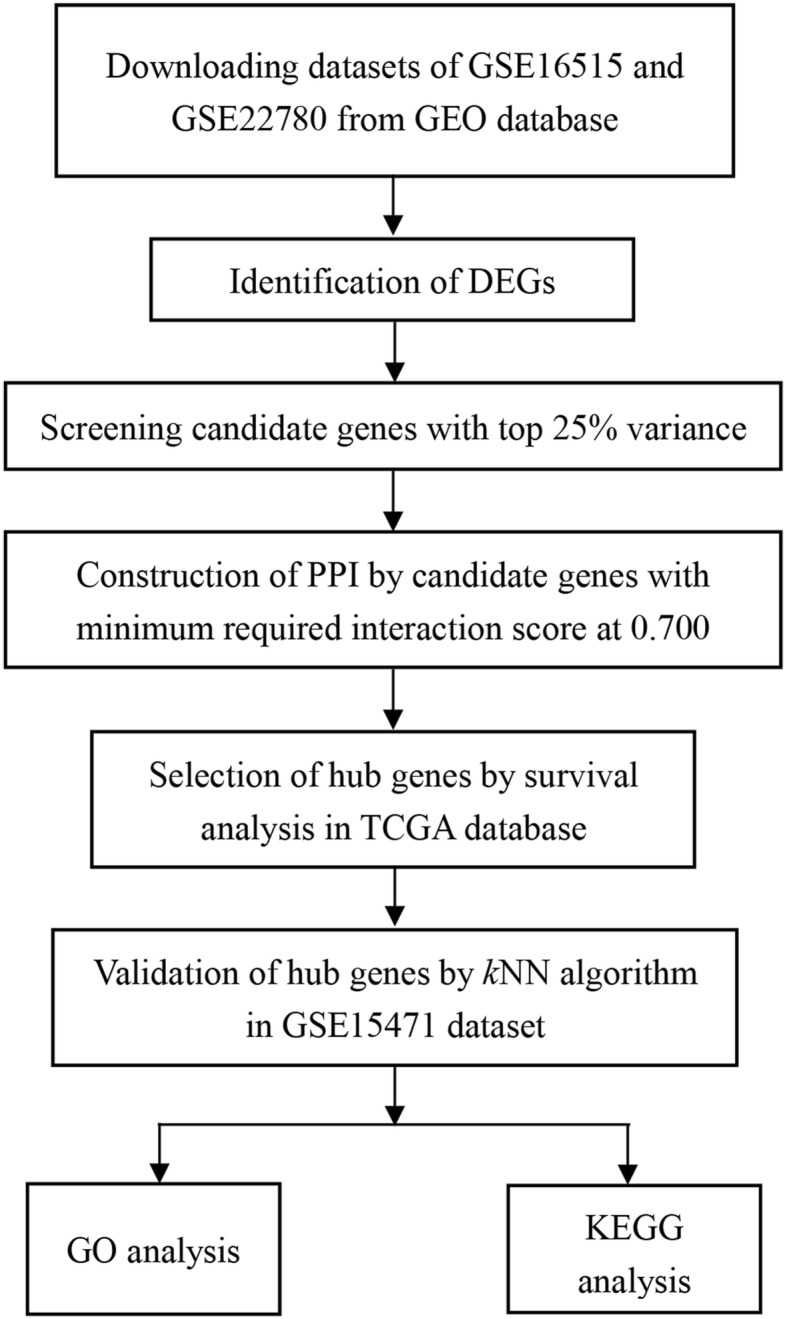
Flow diagram of the analysis procedure: data collection, analysis, hub gene selection and validation

A workflow of this study was shown in Fig. 1. Datasets in our study were firstly searched in GEO database (http://www.ncbi.nlm.nih.gov/geo/) by using these keywords “pancreatic/pancreas” + “tumor/cancer” + “normal” + “GPL570,” and 165 datasets were obtained until June 20th, 2018. Then these datasets were further screened as following criteria: (1) Samples were from human pancreatic tissues. (2) Samples were not interfered with any other treatments. Finally, three datasets, GSE16515 [20], GSE22780, and GSE15471 [21], were included in our study for further analysis.

All the datasets were performed by Affymetrix Human Genome U133 Plus 2.0 Array (Affymetrix, Santa Clara, CA, USA). GSE16515 dataset included 36 malignant pancreatic samples and 16 normal pancreatic samples, while the corresponding numbers in GSE22780 dataset were 8 and 8. In order to obtain sample balance, combination of GSE16515 and GSE22780 was used as training set to determine hub genes. Besides, raw expression data of GSE15471 was downloaded from GEO, also performed by Affymetrix Human Genome U133 Plus 2.0 Array. It composed of 39 normal and 39 malignant pancreatic samples, and served as testing set.

Firstly, the quality of all the datasets were detected with “affyPLM” package in R, herein FitPLM weight, residual, relative log expression (RLE), normalized unscaled standard errors (NUSE), and RNA degradation images were evaluated. Then robust multiarray averaging (RMA) with “affy” package was used to do the background correction and normalization. Before subsequent hub gene selection in training set, empirical Bayes framework with “sva” package in R was used to adjust the batch effects between these two datasets.

In addition, we also downloaded RNA-sequencing data of pancreatic cancer from The Cancer Genome Atlas (TCGA) database (https://cancergenome.nih.gov/), and all the raw data were also converted into gene symbol expression matrix by R software and Perl software.

### Differentially expressed genes screening

Herein, “limma” package was used to detect differentially expressed genes (DEGs) between malignant pancreatic samples and normal samples in training set with the threshold of adj.*P* value < 0.01 and absolute log2-based fold change > 1.

### Candidate gene selection

Variance of every DEGs in different samples were calculated and sorted by descending order, and the top 25% results were selected. Then, 181 genes bearing top 25% variance were uploaded in Search Tool for the Retrieval of Interacting Genes (STRING) database (https://string-db.org/), and PPI network was constructed [22] by setting minimum required interaction score at 0.700. Then a plug-in Cytohubba in Cytoscape [23] was used to further screen candidate genes. Herein, degree algorithm was applied and the screening criterion was degree > 5.

### Hub gene screening by survival and cox regression analyses in TCGA

Candidate genes were further screened by survival analysis and cox regression analysis in TCGA database with “survival” package. Genes with *P* value less than 0.05 both in survival analysis and cox analysis were further screened as hub genes.

### Gene ontology annotation and pathway analyses of candidate genes

In order to depict the biological function of candidate genes, gene ontology (GO) biological process enrichments were performed through Database for Annotation, Visualization and Integrated Discovery (DAVID) (https://david.ncifcrf.gov/) [24, 25]. And the visualization of GO results was performed by “GOplot” package in R.

### Validation of hub genes by supervised learning methods

In order to verify whether these hub genes were “real hub genes” to discriminate tumor and normal samples, *k*NN algorithm in “class” package and random forest algorithm in “randomForest” package were performed. The accuracy was used to evaluate the predictive results. Herein, random forest algorithm was rerun for 100 times, and the mean value of the accuracies was calculated finally.

## Results

### Identification of DEGs

**Fig. 2:**
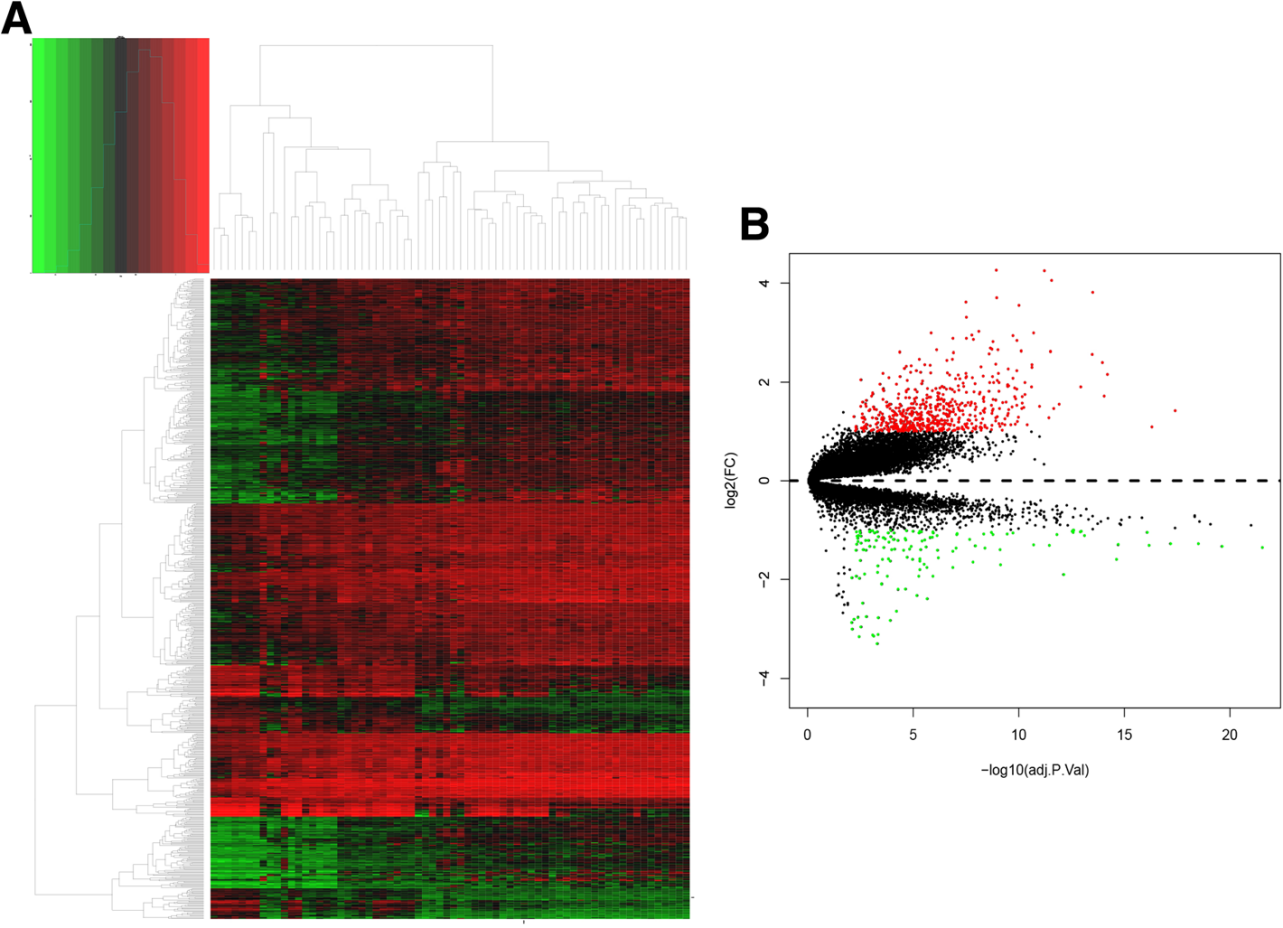
Identification of differentially expressed genes (DEGs). Note: **a** heatmap for all the genes. **b** Volcano map for DEGs selection, red dots represented upregulated genes and green dots represented downregulated genes

After the quality control of GSE16515 and GSE22780 datasets, these two profiles were suitable for subsequent analyses. And all the raw probe expression data were converted into gene expression data finally. The heat map of all the gene expressions in training set was shown in Fig. 2a. After background correction and normalization as well as batch effects adjustment, 724 DEGs were determined with the threshold of adj.*P* value < 0.01 and absolute log2-based fold change > 1 (Additional file 1). Among all the DEGs, there were 591 upregulated genes and 133 downregulated genes, and the volcano map for DEGs selection was shown in Fig. 2b.

### Determination of candidate gene*s*

**Fig. 3:**
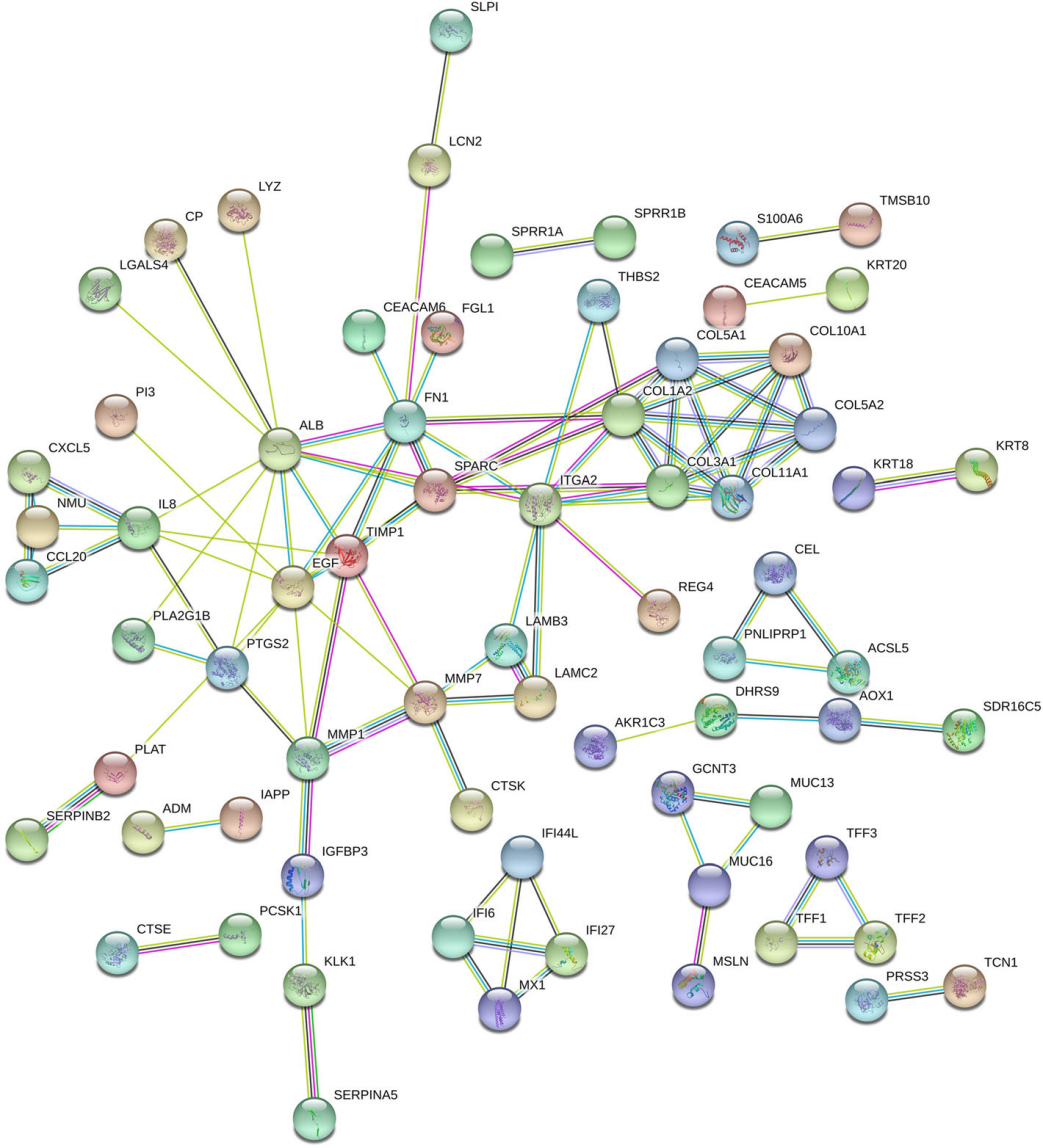
PPI network constructed by 181 candidate genes with minimum required interaction score at 0.700

Variance analyses of 724 DEGs were further performed in all the 68 different samples, and 181 candidate genes with top 25% variance were screened (shown in Additional file 2). Subsequently, all the 181 candidate genes were uploaded to STRING database, and PPI network was constructed with minimum required interaction score at 0.700. After elimination of disconnected node in the network, there were 175 nodes and 102 edges in this PPI network (Fig. 3). Finally, 11 genes (*ALB*, *EGF*, *FN1*, *ITGA2*, *COL1A2*, *SPARC*, *COL3A1*, *TIMP1*, *COL5A1*, *COL11A1*, and *MMP7*) with degree > 5 were screened as candidate genes.

### Selection of hub genes by survival and cox analyses

**Fig. 4:**
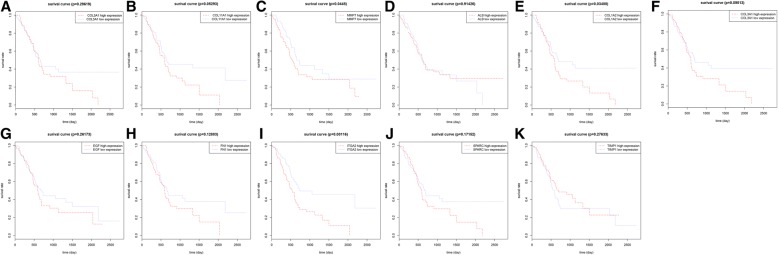
Survival analysis to select hub genes in the TCGA dataset. Note: **a** *COL5A1*, **b** *COL11A1*, **c** *MMP7*, **d** *ALB*, **e** *COL1A2*, **f** *COL3A1*, **g** *EGF*, **h** *FN1*, **i** *ITGA2*, **j** *SPARC*, **k***TIMP1*

There were 178 pancreatic cancer samples and 4 normal samples in TCGA database. In survival analysis, two groups were defined, one is high expression group (expressions greater than mean expression of the gene) and the other one is low expression group (expressions lower than mean expression of the gene). After survival analyses of 11 candidate genes, 3 genes (*MMP7*, *COL1A2*, and *ITGA2*) had significant difference of survival time between these two groups (Fig. 4). As for cox regression analysis, two genes (*MMP7* and *ITGA2*) bear significant difference between alive and death patients. Therefore, *MMP7* and *ITGA2* were further screened as hub genes for further analysis.

### Functional annotation and pathway enrichment

**Fig. 5:**
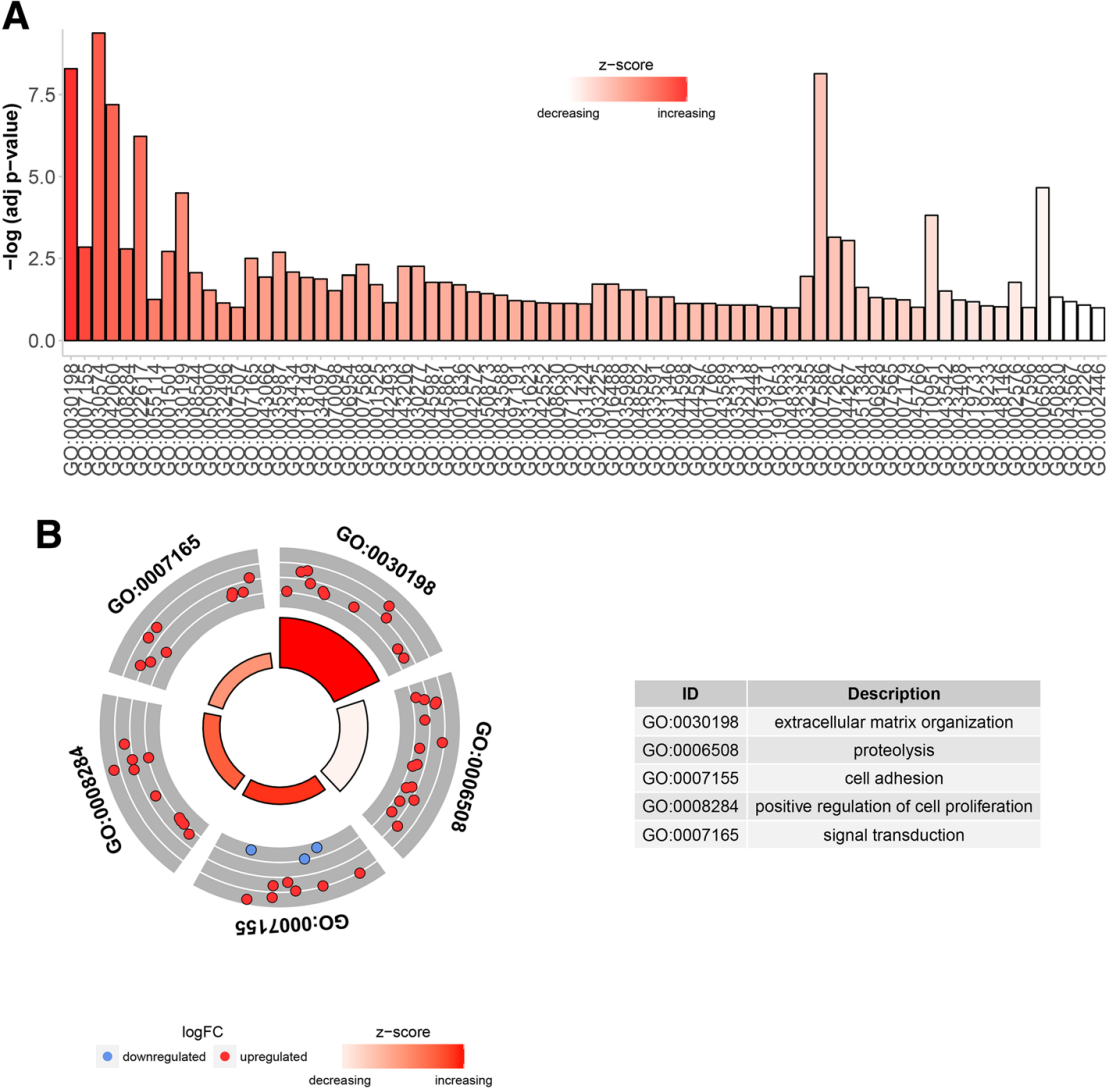
GO annotation for all the 181 candidate genes. Note: **a** Expressions of every GO clusters. **b** Functional annotation of top 5 GO enriched the most genes

GO enrichment results showed that 181 genes were participated in 75 different biological process, and genes in GO:0030198 implicated in extracellular matrix organization exhibited the most significantly upregulated expressions (Fig. 5a). In Fig. 5b, the biological processes of top 5 GO terms enriched the most genes were shown, of which GO:0007165 enriched 22 genes ranked as the first with the biological process of signal transduction. GO enrichment of two hub genes demonstrated that these hub genes mainly participated in the regulation of cell adhesion, transforming growth factor beta receptor signaling pathway and extracellular matrix organization or disassembly (Table 1).

**Table 1:** Functional annotation of two hub genes *ITGA2* and *MMP7*

Genes	GO number	Biological process
*ITGA2*	GO:0045987	Positive regulation of smooth muscle contraction
	GO:0033591	Response to l-ascorbic acid
	GO:0031346	Positive regulation of cell projection organization
	GO:0043589	Skin morphogenesis
	GO:0048333	Mesodermal cell differentiation
	GO:0030198	Extracellular matrix organization
	GO:0007155	Cell adhesion
	GO:0042493	Response to drug
	GO:0007596	Blood coagulation
	GO:0007565	Female pregnancy
*MMP7*	GO:0006508	Proteolysis
	GO:0030574	Collagen catabolic process
	GO:0022617	Extracellular matrix disassembly
	GO:0007568	Aging

### Prediction of pancreatic cancer by hub genes

Herein, *k*NN and random forest algorithms were applied to detect whether these two hub genes could correctly distinguish malignant samples from normal samples. We can see from Table 2 that hub genes selected by method 1 (the method performed in this study) bear the highest predictive accuracy, which reached to almost 93.59% by using *k*NN method. As for random forest algorithm, the mean predictive accuracy was 81.31% after rerunning the method for 100 times. Furthermore, predictive accuracies of different hub genes selected by other methods were compared, and the results were listed in Table 2. Conclusion could be drawn from Table 2 that method 1 as proposed in this study had highly predictive accuracies in both *k*NN and random forest algorithms.

**Table 2:** Comparison of predictive accuracy resulted from different screening methods

Minimum required interaction score	Methods	Hub genes	*k*	Accuracy of *k*NN algorithm	Mean accuracy of random forest algorithm (rerun 100 times)
0.700	Method 1: 724 DGEs-181 candidate genes-genes bearing top 10 degrees in PPI-2 hub genes by survival analysis and cox analysis	*MMP7*, *ITGA2*	2	78.21%	81.31%
			5	84.62%	
			10	87.18%	
			23	92.31%	
			27	93.59%	
	Method 2: 724 DGEs-181 candidate genes-genes bearing top 10 degrees in PPI	*ALB*, *EGF*, *FN1*, *ITGA2*, *COL1A2*, *SPARC*, *COL3A1*, *TIMP1*, *COL5A1*, *COL11A1*, *MMP7*	2	79.49%	83.54%
			4	70.51%	
			6	76.92%	
			9	78.20%	
			13	80.77%	
			15	88.46%	
			18	83.33%	
	Method 3: 724 DGEs-genes bearing top 10 degrees in PPI-2 hub genes by survival analysis and cox analysis	*TOP2A*, *MAD2L1*	2	65.38%	69.82%
			5	69.23%	
			8	65.38%	
			12	66.67%	
			23	67.95%	
	Method 4: 724 DGEs-genes bearing top 10 degrees in PPI	*CCNB1*, *CCNA2*, *MAD2L1*, *TOP2A*, *UBE2C*, *CDC20*, *TTK*, *MELK*, *BUB1B*, *NDC80*	2	70.51%	74.81%
			5	71.80%	
			8	76.92%	
			13	75.64%	
			23	74.36%	
0.400	Method 5: 724 DGEs-181 candidate genes-genes bearing top 10 degrees in PPI-1 hub genes by survival analysis and cox analysis	*ITGA2*	2	74.36%	69.23%
			5	80.77%	
			10	80.77%	
			14	80.77%	
			18	82.05%	
			22	85.90%	
	Method 6: 724 DGEs-181 candidate genes-genes bearing top 10 degrees in PPI	*ALB, EGF, ITGA2, FN1, COL1A2, TIMP1, MMP1, COL3A1, PTGS2, CEL*	2	82.05%	83.72%
			4	71.80%	
			6	79.49%	
			10	75.64%	
			13	74.36%	
			18	73.08%	
	Method7:724 DGEs-genes bearing top10 degrees in PPI-2 hub genes by survival analysis and cox analysis	*TOP2A*, *MAD2L1*	2	65.38%	69.82%
			5	69.23%	
			8	65.38%	
			12	66.67%	
			23	67.95%	
	Method8:724 DGEs-genes bearing top 10 degrees in PPI	*ALB*, *GAPDH*, *EGF*, *TOP2A*, *CCNB1*, *NDC80*, *CCNA2*, *CDC20*, *UBE2C*, *BUB1B*, *MAD2L1*, *TTK*, *OIP5*, *KIF11*	2	71.79%	73.05%
			6	73.08%	
			11	69.23%	
			15	70.51%	
			22	73.08%	

Method 1: Identification of DEGs → screening candidate genes with top 25% variance → construction of PPI by candidate genes with minimum required interaction score at 0.700, and further screen candidate genes with top 10 degrees in PPI → Selection of hub genes by survival and cox analyses in TCGA database

Method 2: Identification of DEGs → screening candidate genes with top 25% variance → construction of PPI by candidate genes with minimum required interaction score at 0.700, and further identification of hub genes bearing top 10 degrees in PPI

Method 3: Identification of DEGs → construction of PPI by candidate genes with minimum required interaction score at 0.700, and further screen candidate genes with top 10 degrees in PPI → selection of hub genes by survival and cox analyses in TCGA database

Method 4: Identification of DEGs → construction of PPI by candidate genes with minimum required interaction score at 0.700, and further identification of hub genes bearing top 10 degrees in PPI

Method 5: Identification of DEGs → screening candidate genes with top 25% variance → construction of PPI by candidate genes with minimum required interaction score at 0.400, and further screen candidate genes with top 10 degrees in PPI → selection of hub genes by survival and cox analyses in TCGA database

Method 6: Identification of DEGs → screening candidate genes with top 25% variance → construction of PPI by candidate genes with minimum required interaction score at 0.400, and further identification of hub genes bearing top 10 degrees in PPI

Method 7: Identification of DEGs → construction of PPI by candidate genes with minimum required interaction score at 0.400, and further screen candidate genes with top 10 degrees in PPI → selection of hub genes by survival and cox analyses in TCGA database

Method 8: Identification of DEGs → construction of PPI by candidate genes with minimum required interaction score at 0.400, and further identification of hub genes bearing top 10 degrees in PPI

## Discussion

Compared with other cancers, the occurrence of pancreatic cancer is relatively rare; however, it is still a lethal disease with poor prognosis. Until now, there still lacks effective therapies against pancreatic cancer, and many novel therapies are in the experimental stage. Therefore, it is important to find some potential hub genes playing crucial roles in regulating cancer occurrence and progression, which may become key targets in the treatment of pancreatic cancer in the future. In addition, these hub genes effectively differing cancer tissues from normal samples may provide novel auxiliary evidence in pancreatic cancer diagnosis. It is demonstrated that pancreatic cancer results from the accumulation of acquired mutations, which may lead to the upregulation of some oncogenes and downregulation of some tumor-suppressing genes and genomic maintenance genes [4]. Therefore, there might exist some DEGs between normal and tumor samples, and these DEGs may play important roles in regulating tumor occurrence, development, and progression. In the present study, two genes *ITGA2* and *MMP7* were screened from DEGs as hub genes by using a series of bioinformatics methods, and they could discriminate normal samples and tumor samples.

The matrix metalloproteinase (MMPs) is a family of enzymes, bearing the capability to cleave extracellular matrix substrates [26], as well as promotes the release of pro-TNF-α, Fas ligand, and some cytokines in various cancers cells [27]. One previous study has experimentally demonstrated that genes in matrix metallopeptidase family, collagen family, and integrin family were upregulated in pancreatic cancer, and they may correlate with cancer activity and poor prognosis [28]. MMPs also involved in proliferative, migrating, and differentiated processes in cells [29]. The interaction between MMPs and extracellular ligand induced a series of signaling cascade, and thus led to the functional regulation of intracellular and extracellular activities. The expression of MMP7 has been reported to be upregulated in several kinds of cancer, including colon cancer [27], pancreatic cancer [30], breast cancer [31], gastric cancer [32], and esophageal cancer [33]. One study has demonstrated that multiplex detection of pancreatic biomarkers CA19-9, MMP7, and MUC4 in sera samples were of high sensitivity, which may act as the critical biomarker in diagnosis of pancreatic cancer [34]. Another study compared tumor tissues with healthy control samples revealed that MMP7 was highly predictive for advanced stage of pancreatic cancer, which strongly associated with N1 status, T3/T4 stage, moderate/poor differentiation, and perineural invasion [35]. It has been reported that Stat3 was a critical factor to facilitate precursor formation and enforced MMP7 expression in pancreatic cancer cells, while MMP7 level was correlated with metastasis and survival in pancreatic cancer patients [36].

ITGA2 encoding by *ITGA2* gene is the alpha subunit of the transmembrane receptor integrin, and it mainly exerts the adhesive roles in cell-cell interaction, also promotes the generation and adhesion of newly synthesized extracellular matrix [37, 38]. The polymorphisms of *ITGA2* gene was related to the poor survival of nasopharyngeal carcinoma [39]. *ITGA2* gene was reported to play migrating roles in colon cancer cells [40], and it expressed in colorectal cancer with liver metastasis tissues but absent in normal tissue [41]. In addition, epigenetic modifications such as DNA methylation were also important in tumorigenesis, and hypomethylation of *ITGA2* with high gene expression was associated with poor survival in pancreatic cancer patients [42]. One research has found that ITGA2 was overexpressed in a variety of gastric cancer patients mainly playing pro-survival roles, and the blockage of ITGA2 could induce apoptosis and inhibit cell migration in gastric cancer [43]. Another research in gastric cancer revealed that HMGA2, FOXL2, and ITGA2 were increased in metastatic lymph nodes and distant metastases in gastric cancer, and suppressing the HMGA2-FOXL2-ITGA2 pathway could serve as a new strategy in further treatment in gastric cancer [44]. The transcriptional co-activators yes-associated protein (YAP) was considered as oncogene in many types of cancer; ITGA2 stimulating YAP activity was associated with unfavorable survival of pancreatic cancer patients [45].

In order to validate whether these genes were real hub genes, another mRNA expression profile GSE15471 from GEO database was utilized as testing set. Herein, *k*NN and random forest algorithms were performed to detect whether these hub genes could successfully distinguish tumor tissues from normal samples. We can see from Table 2 that hub genes selected by method 1 in this study represented the highest accuracy reaching to 94% approximately with 2.56% false negative and 3.84% false positive. In the cases of differing from tumor and normal samples, reduction of false negative results was more important than the reduction of false positive result. Since false negative results may lead to wrongly diagnose pancreatic cancer as normal condition, it may result in the delay of timely treatment, and further lead to more serious progression of disease as well as more waste of medical resources and costs. Bedsides, random forest algorithm also represented highly predictive accuracy of 81.31% after rerun for 100 times of method 1. Therefore, method 1 bear highly predictive accuracies in both of the two methods, and it could be inferred that these two hub genes were real hub genes, which could successfully discriminate normal and tumor samples. Another interesting result could be found in Table 2 that selection of genes with top 25% variance obviously increased the predictive accuracy from 70 to 94% (method 1 vs. method 3).

In addition, we can choose different minimum required interaction score when constructing PPI network. Minimum required interaction score is a threshold providing a score for each interactive pair, which is computed as the joint probability from different evidence (e.g., protein interaction, fusion, co-expression, text mining). Higher score may represent more confident interaction while lower score may lead to more false positives [22]. In order to elucidate whether setting different minimum required interaction score may have influence on hub gene selection, predictive accuracy was compared (shown in Table 2). It can be found that higher minimum required interaction score led to much higher predictive accuracies; method 5 bear the highest accuracy of 85% while the predictive accuracy of method 1 could reach to almost 95% by using *k*NN method.

Hub genes screened in this study were rational. Firstly, all the candidate genes and these two hub genes were closely correlated with the progression of tumor. As shown in GO enrichment, most of the candidate genes were implicated in the biological process of extracellular matrix, cell adhesion, cell proliferation, and signal transduction; they play important role in the progression of cancers. Moreover, both of the hub genes were implicated in the regulation of tumor microenvironment, including the regulation of tumor cells, stroma cells, extracellular matrix (ECM), and some extracellular molecules like cytokines as well as chemokines. It has been demonstrated that microenvironment was usually dysregulated and disorganized in cancer cells. Thus, disordered microenvironment may be favorable to tumor proliferation, progression, invasion and metastasis, and exert drug-hampering roles [46, 47], and now some treatment strategies have focused on the regulation of tumor microenvironment. Since pancreatic cancer was featured as uncontrolled and malignant invasion and migration, therefore we can infer that these hub genes implicated in tumor microenvironment might be core meditators in pancreatic cancer diagnosis and therapy. Secondly, two supervised learning methods were performed, and both of the predictive results of these two hub genes were good with lower false negative in discriminating tumor samples from normal samples.

However, there also exist some limitations in our study. Firstly, the number of samples in our study is not too much. According to the dataset screening criteria, three datasets were included in our study. There were 146 samples totally, of which 68 were training set and 78 were testing set. In the future, with more and more investigations about pancreatic cancer would be performed, more samples should be included. Secondly, in this study, we mainly focused on the genes in the pancreatic tissue not the genes from circulating tumor cells (CTC) nor circulating tumor DNA (ctDNA) in peripheral blood, since the genes in tissue are more accurate to analyze the important biomarkers. Moreover, the datasets about peripheral blood in GEO database are not enough to do the same research. In the future, the microarray analysis of DNA in peripheral blood of pancreatic cancer patients should be further proposed. Thirdly, in our study, all the hub genes were screened and validated only by bioinformatics method, and further exploration of the biological functions and molecular mechanisms of these hub genes both in vitro and in vivo are needed to be fulfilled.

## Conclusions

In summary, we conducted a series of bioinformatics methods to find DEGs, further screened and validated hub genes. These two hub genes, *ITGA2* and *MMP7*, may act as potential diagnostic and therapeutic biomarkers in pancreatic cancer patients. This study provides several useful hub genes for future in vitro and in vivo investigations of their molecular mechanisms in pancreatic cancer diagnosis and therapy. And profile data mining by bioinformatics analysis is an available method to find potential diagnostic and prognostic biomarkers systematically. Nevertheless, further molecular mechanisms investigations by biological experiments are still needed to be verified in pancreatic cancer cells.

## Additional files

Additional file 1: Identification of 724 DEGs. (XLS 134 kb)

Additional file 2: Identification of 181 candidate genes with top 25% variance. (XLS 36 kb)

## References

[1] Li C, Heidt DG, Dalerba P, Burant CF, Zhang L, Adsay V, Wicha M, Clarke MF, Simeone DM (2007). Identification of pancreatic cancer stem cells. Cancer Res.

[2] Rahib L, Smith BD, Aizenberg R, Rosenzweig AB, Fleshman JM, Matrisian LM (2014). Projecting cancer incidence and deaths to 2030: the unexpected burden of thyroid, liver, and pancreas cancers in the United States. Cancer Res.

[3] Korc M (2007). Pancreatic cancer-associated stroma production. Am J Surg.

[4] Kleeff J, Korc M, Apte M, La Vecchia C, Johnson CD, Biankin AV, Neale RE, Tempero M, Tuveson DA, Hruban RH, Neoptolemos JP (2016). Pancreatic cancer. Nat Rev Dis Primers.

[5] Neoptolemos JP, Palmer DH, Ghaneh P, Psarelli EE, Valle JW, Halloran CM, Faluyi O, O'Reilly DA, Cunningham D, Wadsley J (2017). Comparison of adjuvant gemcitabine and capecitabine with gemcitabine monotherapy in patients with resected pancreatic cancer (ESPAC-4): a multicentre, open-label, randomised, phase 3 trial. Lancet.

[6] Waddell N, Pajic M, Patch AM, Chang DK, Kassahn KS, Bailey P, Johns AL, Miller D, Nones K, Quek K (2015). Whole genomes redefine the mutational landscape of pancreatic cancer. Nature.

[7] Wang L, Zhou W, Zhong Y, Huo Y, Fan P, Zhan S, Xiao J, Jin X, Gou S, Yin T (2017). Overexpression of G protein-coupled receptor GPR87 promotes pancreatic cancer aggressiveness and activates NF-kappaB signaling pathway. Mol Cancer.

[8] Zhong Y, Naito Y, Cope L, Naranjo-Suarez S, Saunders T, Hong SM, Goggins MG, Herman JM, Wolfgang CL, Iacobuzio-Donahue CA (2014). Functional p38 MAPK identified by biomarker profiling of pancreatic cancer restrains growth through JNK inhibition and correlates with improved survival. Clin Cancer Res.

[9] Khan MA, Zubair H, Srivastava SK, Singh S, Singh AP (2015). Insights into the role of microRNAs in pancreatic cancer pathogenesis: potential for diagnosis, prognosis, and therapy. Adv Exp Med Biol.

[10] Dillhoff M, Liu J, Frankel W, Croce C, Bloomston M (2008). MicroRNA-21 is overexpressed in pancreatic cancer and a potential predictor of survival. J Gastrointest Surg.

[11] Li J, Wu H, Li W, Yin L, Guo S, Xu X, Ouyang Y, Zhao Z, Liu S, Tian Y (2016). Downregulated miR-506 expression facilitates pancreatic cancer progression and chemoresistance via SPHK1/Akt/NF-kappaB signaling. Oncogene.

[12] Ji Qing, Hao Xinbao, Zhang Min, Tang Wenhua, Yang Meng, Li Ling, Xiang Debing, DeSano Jeffrey T., Bommer Guido T., Fan Daiming, Fearon Eric R., Lawrence Theodore S., Xu Liang (2009). MicroRNA miR-34 Inhibits Human Pancreatic Cancer Tumor-Initiating Cells. PLoS ONE.

[13] Edgar R, Domrachev M, Lash AE (2002). Gene expression omnibus: NCBI gene expression and hybridization array data repository. Nucleic Acids Res.

[14] Cancer Genome Atlas Research N, Weinstein JN, Collisson EA, Mills GB, Shaw KR, Ozenberger BA, Ellrott K, Shmulevich I, Sander C, Stuart JM (2013). The Cancer Genome Atlas Pan-Cancer analysis project. Nat Genet.

[15] Rajamani D, Bhasin MK (2016). Identification of key regulators of pancreatic cancer progression through multidimensional systems-level analysis. Genome Medicine.

[16] Chuang HY, Lee E, Liu YT, Lee D, Ideker T (2007). Network-based classification of breast cancer metastasis. Mol Syst Biol.

[17] Sivakumar S, de Santiago I, Chlon L, Markowetz F (2017). Master regulators of oncogenic KRAS response in pancreatic cancer: an integrative network biology analysis. PLoS Medicine / Public Library of Science.

[18] Muzumdar MD, Chen PY, Dorans KJ, Chung KM, Bhutkar A, Hong E, Noll EM, Sprick MR, Trumpp A, Jacks T (2017). Survival of pancreatic cancer cells lacking KRAS function. Nat Commun.

[19] Wolfgang CL, Herman JM, Laheru DA, Klein AP, Erdek MA, Fishman EK, Hruban RH (2013). Recent progress in pancreatic cancer. CA Cancer J Clin.

[20] Pei H, Li L, Fridley BL, Jenkins GD, Kalari KR, Lingle W, Petersen G, Lou Z, Wang L (2009). FKBP51 affects cancer cell response to chemotherapy by negatively regulating Akt. Cancer Cell.

[21] Badea L, Herlea V, Dima SO, Dumitrascu T, Popescu I (2008). Combined gene expression analysis of whole-tissue and microdissected pancreatic ductal adenocarcinoma identifies genes specifically overexpressed in tumor epithelia. Hepatogastroenterology.

[22] Szklarczyk D, Franceschini A, Wyder S, Forslund K, Heller D, Huerta-Cepas J, Simonovic M, Roth A, Santos A, Tsafou KP (2015). STRING v10: protein-protein interaction networks, integrated over the tree of life. Nucleic Acids Res.

[23] Chin CH, Chen SH, Wu HH, Ho CW, Ko MT, Lin CY (2014). cytoHubba: identifying hub objects and sub-networks from complex interactome. BMC Syst Biol.

[24] Ashburner M, Ball CA, Blake JA, Botstein D, Butler H, Cherry JM, Davis AP, Dolinski K, Dwight SS, Eppig JT (2000). Gene ontology: tool for the unification of biology. The gene ontology consortium. Nat Genet.

[25] Dennis G, Sherman BT, Hosack DA, Yang J, Gao W, Lane HC, Lempicki RA (2003). DAVID: database for annotation, visualization, and integrated discovery. Genome Biol.

[26] Van Wart HE, Birkedal-Hansen H (1990). The cysteine switch: a principle of regulation of metalloproteinase activity with potential applicability to the entire matrix metalloproteinase gene family. Proc Natl Acad Sci U S A.

[27] Jang B, Jung H, Choi S, Lee YH, Lee S-T (2017). Oh E-S: Syndecan-2 cytoplasmic domain up-regulates matrix metalloproteinase-7 expression via the protein kinase Cgamma-mediated FAK/ERK signaling pathway in colon cancer. J Biol Chem.

[28] Moffitt RA, Marayati R, Flate EL, Volmar KE, Loeza SG, Hoadley KA, Rashid NU, Williams LA, Eaton SC, Chung AH (2015). Virtual microdissection identifies distinct tumor- and stroma-specific subtypes of pancreatic ductal adenocarcinoma. Nat Genet.

[29] Malemud CJ (2006). Matrix metalloproteinases (MMPs) in health and disease: an overview. Front Biosci.

[30] Chen SH, Hung WC, Wang P, Paul C, Konstantopoulos K (2013). Mesothelin binding to CA125/MUC16 promotes pancreatic cancer cell motility and invasion via MMP-7 activation. Sci Rep.

[31] Lin HY, Sun SM, Lu XF, Chen PY, Chen CF, Liang WQ, Peng CY (2017). CCR10 activation stimulates the invasion and migration of breast cancer cells through the ERK1/2/MMP-7 signaling pathway. Int Immunopharmacol.

[32] Xu J, E C, Yao Y, Ren S, Wang G, Jin H (2016). Matrix metalloproteinase expression and molecular interaction network analysis in gastric cancer. Oncol Lett.

[33] Juchniewicz A, Kowalczuk O, Milewski R, Laudanski W, Dziegielewski P, Kozlowski M, Niklinski J (2017). MMP-10, MMP-7, TIMP-1 and TIMP-2 mRNA expression in esophageal cancer. Acta Biochim Pol.

[34] Banaei Nariman, Foley Anne, Houghton Jean Marie, Sun Yubing, Kim Byung (2017). Multiplex detection of pancreatic cancer biomarkers using a SERS-based immunoassay. Nanotechnology.

[35] Wang SC, Parekh JR, Porembka MR, Nathan H, D'Angelica MI, DeMatteo RP, Fong Y, Kingham TP, Jarnagin WR, Allen PJ (2016). A pilot study evaluating serum MMP7 as a preoperative prognostic marker for pancreatic ductal adenocarcinoma patients. J Gastrointest Surg.

[36] Fukuda A, Wang SC, JPt M, Folias AE, Liou A, Kim GE, Akira S, Boucher KM, Firpo MA, Mulvihill SJ, Hebrok M (2011). Stat3 and MMP7 contribute to pancreatic ductal adenocarcinoma initiation and progression. Cancer Cell.

[37] Bergelson JM, St John N, Kawaguchi S, Chan M, Stubdal H, Modlin J, Finberg RW (1993). Infection by echoviruses 1 and 8 depends on the alpha 2 subunit of human VLA-2. J Virol.

[38] Graham KL, Halasz P, Tan Y, Hewish MJ, Takada Y, Mackow ER, Robinson MK, Coulson BS (2003). Integrin-using rotaviruses bind alpha2beta1 integrin alpha2 I domain via VP4 DGE sequence and recognize alphaXbeta2 and alphaVbeta3 by using VP7 during cell entry. J Virol.

[39] Ban EZ, Lye MS, Chong PP, Yap YY, Lim SYC, Abdul Rahman H (2018). Association of hOGG1 Ser326Cys, ITGA2 C807T, TNF-A -308G>a and XPD Lys751Gln polymorphisms with the survival of Malaysian NPC patients. PLoS One.

[40] Ferraro A, Boni T, Pintzas A (2014). EZH2 regulates cofilin activity and colon cancer cell migration by targeting ITGA2 gene. PLoS ONE [electronic Resource].

[41] Yang Q, Bavi P, Wang JY, Roehrl MH (2017). Immuno-proteomic discovery of tumor tissue autoantigens identifies olfactomedin 4, CD11b, and integrin alpha-2 as markers of colorectal cancer with liver metastases. J Proteome.

[42] Nones K, Waddell N, Song S, Patch AM, Miller D, Johns A, Wu J, Kassahn KS, Wood D, Bailey P (2014). Genome-wide DNA methylation patterns in pancreatic ductal adenocarcinoma reveal epigenetic deregulation of SLIT-ROBO, ITGA2 and MET signaling. Int J Cancer.

[43] Chuang YC, Wu HY, Lin YL, Tzou SC, Chuang CH, Jian TY, Chen PR, Chang YC, Lin CH, Huang TH, et al. Blockade of ITGA2 induces apoptosis and inhibits cell migration in gastric Cancer. Biol Proced Online. 2018;20:10. 10.1186/s12575-018-0073-x PMC5928594 29743821

[44] Dong J, Wang R, Ren G, Li X, Wang J, Sun Y, Liang J, Nie Y, Wu K, Feng B (2017). HMGA2-FOXL2 Axis regulates metastases and epithelial-to-mesenchymal transition of chemoresistant gastric cancer. Clin Cancer Res.

[45] Rozengurt E, Sinnett-Smith J, Eibl G (2018). Yes-associated protein (YAP) in pancreatic cancer: at the epicenter of a targetable signaling network associated with patient survival. Signal Transduct Target Ther.

[46] Kessenbrock K, Plaks V, Werb Z (2010). Matrix metalloproteinases: regulators of the tumor microenvironment. Cell.

[47] Hanahan D, Coussens LM (2012). Accessories to the crime: functions of cells recruited to the tumor microenvironment. Cancer Cell.

